# Increased expression of ER stress, inflammasome activation, and mitochondrial biogenesis-related genes in peripheral blood mononuclear cells in major depressive disorder

**DOI:** 10.21203/rs.3.rs-3564760/v1

**Published:** 2024-01-12

**Authors:** Soumyabrata Munshi, Ahlam Alarbi, Haixia Zheng, Rayus Kuplicki, Kaiping Burrows, Leandra Figueroa-Hall, Teresa Victor, Robin Aupperle, Sahib Khalsa, Martin Paulus, T. Kent Teague, Jonathan Savitz

**Affiliations:** Laureate Institute for Brain Research; Laureate Institute for Brain Research; Laureate Institute for Brain Research; Laureate Institute for Brain Research; Laureate Institute for Brain Research; Laureate Institute for Brain Research; Laureate Institute for Brain Research; Laureate Institute for Brain Research; Laureate Institute for Brain Research; Laureate Institute for Brain Research, Tulsa, USA; University of Oklahoma School of Community Medicine; Laureate Institute for Brain Research

**Keywords:** Endoplasmic reticulum stress, gene expression, inflammasome, major depressive disorder, mitochondrial biogenesis, peripheral blood mononuclear cells, RT-qPCR

## Abstract

A subset of major depressive disorder (MDD) is characterized by immune system dysfunction, but the intracellular origin of these immune changes remains unclear. Here we tested the hypothesis that abnormalities in the endoplasmic reticulum (ER) stress, inflammasome activity and mitochondrial biogenesis contribute to the development of systemic inflammation in MDD. RT-qPCR was used to measure mRNA expression of key organellar genes from peripheral blood mononuclear cells (PBMCs) isolated from 186 MDD and 67 healthy control (HC) subjects. The comparative CT (2^−ΔΔCT^) method was applied to quantify mRNA expression using *GAPDH* as the reference gene. After controlling for age, sex, BMI, and medication status using linear regression models, expression of the inflammasome (*NLRC4* and *NLRP3*) and the ER stress (*XBP1u*, *XBP1s*, and *ATF4*) genes was found to be significantly increased in the MDD versus the HC group. After excluding outliers, expression of the inflammasome genes was no longer statistically significant but expression of the ER stress genes (*XBP1u*, *XBP1s*, and *ATF4*) and the mitochondrial biogenesis gene, *MFN2*, was significantly increased in the MDD group. *ASC* and *MFN2* were positively correlated with serum C-reactive protein concentrations. The altered expression of inflammasome activation, ER stress, and mitochondrial biogenesis pathway components suggest that dysfunction of these organelles may play a role in the pathogenesis of MDD.

## INTRODUCTION

Some cases of major depressive disorder (MDD) are known to be associated with immune system dysfunction, characterized in part by elevations in circulating inflammatory mediators^1–3^. However, at the cellular level, little is understood about how critical intracellular organelles within immune cells contribute to this pro-inflammatory environment. Here, in peripheral blood mononuclear cells (PBMCs), we measured the expression of representative genes of three structurally and functionally related organelles linked to inflammation – the inflammasomes, the endoplasmic reticula (ER), and the mitochondria^4,5^. A better understanding of the cellular roots of immune cell dysfunction in MDD could ultimately help to identify potential therapeutic targets and lead to new treatment strategies.

The inflammasome is a molecular complex that constitutes an essential component of the host’s defense system, responding to various infectious agents, cellular stress, and tissue damage^6^. Composed of multiple proteins, including pattern recognition receptors (PRRs), nucleotide-binding oligomerization domain (NOD)-like receptors (NLRs) and adaptor molecules like ASC (apoptosis-associated speck-like protein containing a caspase recruitment domain), the inflammasome orchestrates the activation of caspase-1, leading to the maturation and secretion of pro-inflammatory cytokines, such as interleukin-1β (IL-1β) and interleukin-18 (IL-18)^6–9^. Several different subtypes of the inflammasome exist. Although they share certain similarities, they differ in their mode of activation. This study focuses on the NLRC4 and the NLRP3 inflammasomes. The NLRC4 inflammasome consists of the NOD-like receptor family member NLRC4 and the adaptor protein, ASC, and has been reported to be primarily activated by intracellular pathogens^10, 11^. The NLRP3 inflammasome is more versatile and can be activated by various stimuli, including microbial components, endogenous danger signals, and cellular stress^12–14^. It is composed of the NOD-like receptor NLRP3, ASC, and caspase-1.

The extant literature implicates dysregulation of inflammasome activation in the pathogenesis of inflammatory and autoimmune disorders^15, 16^ as well as neurodegenerative diseases^17, 18^. However, the role of the inflammasome in psychiatric disorders such as MDD is less well understood. Numerous preclinical studies have reported that activation of the NLRP3 inflammasome is associated with depressive-like behaviors in rodents^19–24^. Nevertheless, there is a paucity of inflammasome-focused research in clinical populations with only a handful of small (n < 50) human studies published to date. These papers have focused on the NLRP3 inflammasome and are consistent with increased expression of NLRP3, its adaptor proteins, and/or downstream effectors such as IL-18^25–28^. There is also indirect evidence of inflammasome activation in the form of meta-analyses reporting elevated circulating concentrations of IL-1β or IL-1 receptor antagonist (IL1-ra) in MDD^29–31^. To our knowledge the NLRC4 inflammasome has never been examined in MDD.

As alluded to above, inflammasomes are not only assembled during infections and inflammation but under the broader conditions of cellular stress^32, 33^. These stressors include DNA damage, nutrient imbalances, oxidative stress, heat shock and hypoxia, and elicit compensatory, homeostatic responses to restore normal cellular function. One such homeostatic response associated with cellular stress is the unfolded protein response (UPR). The UPR is a cellular signaling pathway that determines the fate of proteins within the organelle that plays a crucial role in protein synthesis and folding, i.e., the ER. Under physiological conditions, the ER functions efficiently to ensure proper folding of nascent polypeptides. However, in the presence of the aforementioned stresses, the ER becomes overwhelmed, leading to the accumulation of unfolded or misfolded proteins^34, 35^.

When confronted with ER stress, the UPR orchestrates a multifaceted response to restore ER homeostasis, characterized by the involvement of three principal arms: the inositol-requiring enzyme 1 (IRE1), the protein kinase RNA-like ER kinase (PERK), and the activating transcription factor 6 (ATF6) pathways^34, 35^. This paper focuses on the IRE1 and PERK arms. IRE1 initiates a signaling cascade through its endoribonuclease activity, splicing the mRNA encoding the transcription factor, X-box binding protein 1 (XBP1). This spliced form of XBP1 enhances the expression of genes encoding ER chaperones and components involved in ER-associated protein degradation, promoting protein folding and clearance^36, 37^. PERK phosphorylates the alpha subunit of eukaryotic translation initiation factor 2 (eIF2α), enhancing the translation of activating transcription factor 4 (ATF4) and hence the UPR^38, 39^. The coordinated activation of these UPR pathway components serves as a mechanism to rectify ER stress^36^. However, chronic ER stress can overwhelm the UPR’s capacity to resolve the underlying protein-folding defects, leading to prolonged UPR activation and ultimately neuronal dysfunction and degeneration, as well as the propagation of toxic protein aggregates^40–42^.

Dysregulation of the UPR has been shown to co-occur with psychological stress^43–46^. Moreover, inflammation, a known risk factor for depression and other psychiatric disorders^1–3^, can activate UPR sensors while conversely, the UPR can regulate the production and release of inflammatory mediators^47–49^. Thus, the literature suggests that perturbations in protein folding could potentially impact not only neuronal plasticity and synaptic function, but also inflammation, inflammasome assembly, and mental health^50^. Nevertheless, there is a paucity of work in this area in the context of psychiatric disorders – to our knowledge, only three publications with small sample sizes (see discussion).

The crosstalk between ER stress, inflammation, and mitochondrial function also plays an important role in cellular homeostasis. Mitochondria physically connect to the ER via specialized contact sites known as the mitochondria-associated ER membrane. ER stress can result in increased cellular energy demand leading to mitochondrial biogenesis, a remodeling process that changes the mitochondrial number, size and/or capacity for energy production^51^. This process of dynamic fission and fusion is regulated by a suite of factors. Two relevant regulatory proteins we focus on in this paper are DNM1L (dynamin 1-like protein, also known as dynamin-related protein 1 or Drp1 encoded by *DNM1L*) and MFN2 (mitofusin-2 encoded by *MFN2*). In response to various types of cellular stress and damage, DNM1L is recruited to the mitochondrial outer membrane where it divides the mitochondrion into smaller fragments, helping to isolate dysfunctional mitochondria^52, 53^. In contrast, MFN2 enables fusion of mitochondria, an adaptation that can also occur during times of high energy demand to promote efficient energy production^52, 54^. To our knowledge, DNM1L has not been studied in the context of MDD, although expression of MFN2 was found to be increased in the PBMCs of individuals with MDD (n = 77) compared with healthy controls (HCs; n = 24)^55^.

In sum, there is emerging evidence from both animal and human studies that inflammasome function, ER stress, and mitochondrial biogenesis are linked to depression. However, there are few existing clinical studies, samples tend to be small, and typically each of these physiological domains is measured in isolation from the others. Here, we quantified the expression of key representative genes from each of these domains in the PBMCs of a well-characterized sample of 186 individuals with MDD and 67 HCs. We hypothesized that individuals with MDD would show increased mRNA expression of ER stress *(XBP1u, XBP1s, ATF4),* inflammasome activation *(NLRC4, NLRP3, ASC),* and mitochondrial biogenesis (*DNM1L, MFN2*) genes.

## PARTICIPANTS AND METHODS

### Participants

Approval for the Tulsa-1000 (T-1000)^56^ study at the Laureate Institute for Brain Research in Tulsa, OK, was obtained from the Western Institutional Review Board. All participants provided written informed consent and all study procedures were carried out in accordance with the principles expressed in the Declaration of Helsinki. Participants were 18–55 years of age and had either no personal history of psychiatric illness (healthy control, HC) or had received a DSM-V diagnosis of MDD (with or without comorbid anxiety) based on the Mini International Neuropsychiatric Inventory (MINI). Depressive symptoms were measured with the Patient Health Questionnaire (PHQ-9)^57^ and early-life stress was measured with the Childhood Trauma Questionnaire (CTQ)^58^. Exclusion criteria are provided in detail elsewhere^56^. In brief, the following factors were exclusionary: comorbid psychiatric disorders (other than anxiety disorders), substance use disorders (except alcohol use disorder), significant or unstable medical conditions (including cardiovascular, gastrointestinal, endocrine, neurological, hematological, rheumatological or metabolic disorders), a history of autoimmune disorders (except treated hypothyroidism), a history of moderate-to-severe traumatic brain injury, a positive urine drug screen, a body mass index (BMI) < 17 or > 38 kg/m^2^.

### Measurement of C-reactive protein and IL-1ra from blood

Serum was isolated following standard laboratory procedures and stored at − 80°C. C-reactive protein (CRP) concentrations were analyzed with the V-PLEX Human Kits on a Meso Quickplex SQ120 instrument (Meso Scale Diagnostics, Maryland, USA). Serum IL-1ra samples were analyzed with the Human Quantikine ELISA Kit (R&D Systems). All samples were run in duplicate. Intra- and inter-assay coefficients of variation were 2.4% and 10.0% (CRP), and 3.1% and 13.2% (IL-1ra), respectively.

### Determination of percent body fat

Percent body fat was measured using an InBody370 Impedance Body Composition Analyzer (InBody Co., Ltd.). This device uses 15 impedance measurements (3 frequencies: 5 kHz, 50 kHz, 250 kHz; five body segments: right arm, left arm, trunk, right leg, left leg) to produce highly accurate composition estimates and has been found to have a high correlation of 0.99 to dual-energy X-ray absorptiometry (DEXA) for lean body mass in a population of normal weight adults. Body mass index (BMI) was calculated using the following formula based on weight and height obtained during medical history of the participants: BMI = weight (kg)/height (m)^2^.

### Preparation of cDNA

PBMCs were isolated from whole blood of the participants and stored frozen in liquid nitrogen, followed by extraction of RNA from the cells following a previously published method^59^. Briefly, frozen PBMCs were quickly thawed in 37°C water-bath followed by addition of cRPMI and centrifugation at 500 × g for 10 min. All items and reagents for cDNA preparation were obtained from Qiagen unless otherwise noted. Cells were then resuspended in RNA lysis buffer (RLT buffer) containing β-mercaptoethanol. The cells were then put on Qiashredder spin columns and centrifuged for 2 min to obtain the lysates, which were then stored frozen at − 80°C. RNA was extracted from the lysates using RNeasy Micro Kits. Any residual DNA was eliminated from the samples by on-column DNase treatment. RNA quality and quantity were assessed on the Bioanalyzer 2100 (Agilent) and Nanodrop (ThermoFisher Scientific). mRNA was reverse transcribed to complementary DNA (cDNA) utilizing the Omniscript Reverse Transcription Kit.

### Reverse transcription – quantitative polymerase chain reaction (RT-qPCR)

All reagents and items used in the RT-qPCR experiments were obtained from ThermoFisher Scientific unless otherwise specified. The cDNAs extracted from the PBMCs of the study participants were stored at − 80°C. After the samples were thawed, a mixture containing 0.5 μL of the cDNA, 10 μL of TaqMan Fast Advanced Master Mix (catalog numbers 4444963 or 4444964), 8.5 μL of UltraPure DNase/RNase-Free Distilled Water (catalog number 10977015) and 1 μL of the respective primers was transferred to the respective wells of a MicroAmp Fast Optical 96-Well Reaction Plate (catalog number 4346907). TaqMan Gene Expression Assay primers (catalog number 4331182) were used for *GAPDH* (assay ID Hs02786624_g1), *NLRC4* (assay ID Hs00892666_m1), *NLRP3* (assay ID Hs00918080_g1), *ASC* or *PYCARD* (assay ID Hs01547324_gH), *DNM1L* (assay ID Hs01552605_m1), *MFN2* (assay ID Hs00208382_m1), *XBP1u* (assay ID Hs02856596_m1), *XBP1s* (assay ID Hs03929085_g1), and *ATF4* (assay ID Hs00909568_g1). All samples were run in triplicate. Corresponding negative control for each plate was set up in the wells for the respective primers following similar steps with the only exception of adding 0.5 μL UltraPure DNase/RNase-Free Distilled Water instead of any cDNA. The well plates were covered with MicroAmp Optical Adhesive Film (catalog number 4311971) and loaded for running under the RT-qPCR program in the QuantStudio 12K Flex Real-Time PCR System. The program was set for 40 cycles in the PCR stage (95 °C for step 1, 60°C for step 2) with a hold stage temperature at 95°C.

### Data analysis

The fold-change values for mRNA expression, normalized to that of *GAPDH*, were obtained utilizing the comparative C_T_ method^60, 61^. Briefly, the Delta C_T_ (ΔC_T_) values for the genes of interest, denoting the difference in the cycle threshold (C_T_) value of the expression of a particular mRNA from the C_T_ value of that of the reference gene (*GAPDH*), were obtained from the DataAssist Software v3.01 (Applied BioSystems). The software calculated the mean ΔC_T_ from the triplicates of each sample for each mRNA. The average across the three measures was always taken as there were no outlier observations among the triplicates. All readings were manually cross-checked for accuracy. The values of the Delta Delta C_T_ (ΔΔC_T_) for the genes were then calculated by subtracting the average ΔC_T_ of the respective genes of the healthy controls from the ΔC_T_ of the respective individual genes of all subjects. Finally, fold-change of the mRNA expression was obtained by applying the following formula: Fold-change = 2^−ΔΔCT^. All data points from the RT-qPCR experiments, including all outliers for the mRNA fold-change values, were used in the final analysis and shown in the figures.

All statistical analyses were run using the RStudio program (https://www.r-project.org). Linear models (‘lm’ function) were used to test for diagnostic differences with age, sex, BMI, and medication status (treated with psychotropic medications versus unmedicated) as covariates. Note that a small number of HC were taking psychotropic medications for purposes other than treatment of depression. Statistical significance was set at *p* < 0.05, two-tailed. Even though we selected the candidate genes *a priori*, and the expression of the genes was correlated with each other, we adopted a conservative approach and performed FDR corrections for multiple comparisons.

Given the heterogeneous nature of MDD, we hypothesized that specific phenotypes (childhood trauma, sleep disturbance, percent body fat, and inflammation) would be associated with the expression of these genes within the MDD group. Therefore, in secondary analyses we correlated the mRNA expression of the genes with CTQ total score, individual items of the PHQ-9, percent body fat, BMI, CRP, and IL-1ra.

## RESULTS

Demographic and clinical differences between the MDD (n = 186) and HC (n = 67) groups are shown in Table 1.

**Figure 1:**
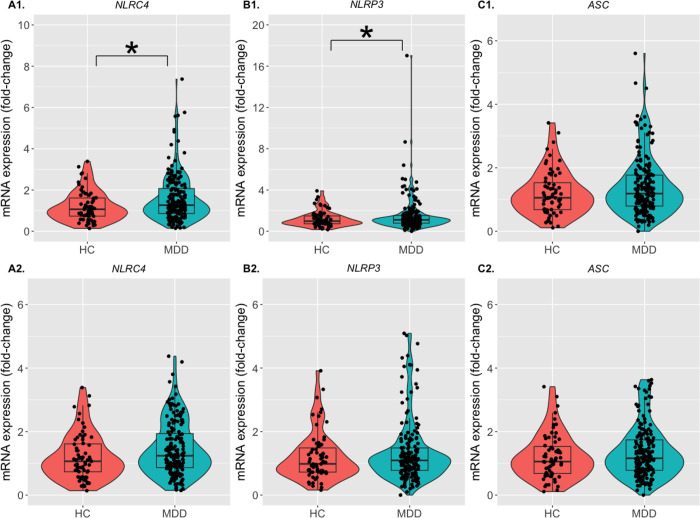
Expression of inflammasome genes in the MDD group compared to the HC group. The violin plots with superimposed box plots and individual data points indicate the fold-change of mRNA expression in the **(A1, A2)** *NLRC4* (left panel), **(B1, B2)** *NLRP3* (middle panel), and **(C1, C2)** *ASC* (right panel) genes for the inflammasome activation. The HC group is represented by neon orange, and the MDD group is represented by teal. The limits of the boxes indicate the 25^th^ and 75^th^ percentile, while the horizontal lines inside the boxes represent the median. The upper and lower limits of the vertical lines from the boxes indicate the 95^th^ percentile of the data. The y-axes indicate the fold-change of the genes’ mRNA expression compared to the respective reference gene (*GAPDH*) in the HC and the MDD groups. Upper panel (**A1, B1, C1**) show all data points, while the lower panel (**A2, B2, C2**) show data points excluding the outliers, which have been defined as data points > 3SD from the mean. The asterisks indicate a statistically significant difference between the two groups (HC vs. MDD). *p < 0.05 after linear regression using age, sex, BMI, and medication status as covariates. HC: Healthy control; MDD: Major depressive disorder.

**Figure 2:**
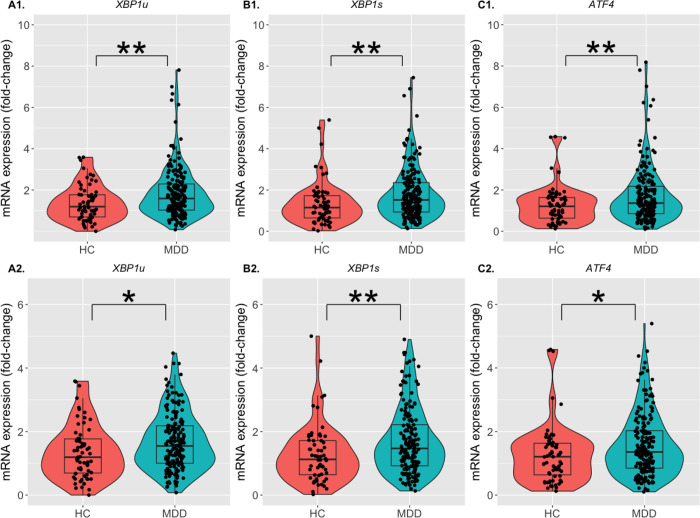
Expression of ER stress genes in the MDD group compared to the HC group. The violin plots with superimposed box plots and individual data points indicating the fold-change of mRNA expression in the **(A1, A2)** *XBP1u* (left panel), **(B1, B2)** *XBP1s* (middle panel), and **(C1, C2)** *ATF4* (right panel) genes for the ER stress. The HC group is represented by neon orange, and the MDD group is represented by teal. The limits of the boxes indicate the 25^th^ and 75^th^ percentile, while the horizontal lines inside the boxes represent the median. The upper and lower limits of the vertical lines from the boxes indicate the 95^th^ percentile of the data. The y-axes indicate the fold-change of the genes’ mRNA expression compared to the respective reference gene (*GAPDH*) in the HC and the MDD groups. Upper panel (**A1, B1, C1**) show all data points, while the lower panel (**A2, B2, C2**) show data points excluding the outliers, which have been defined as data points > 3SD from the mean. The asterisks indicate statistically significant difference between the two groups (HC vs. MDD). *p < 0.05, **p < 0.01 after linear regression using age, sex, BMI, and medication status as covariates. HC: Healthy control; MDD: Major depressive disorder.

**Figure 3:**
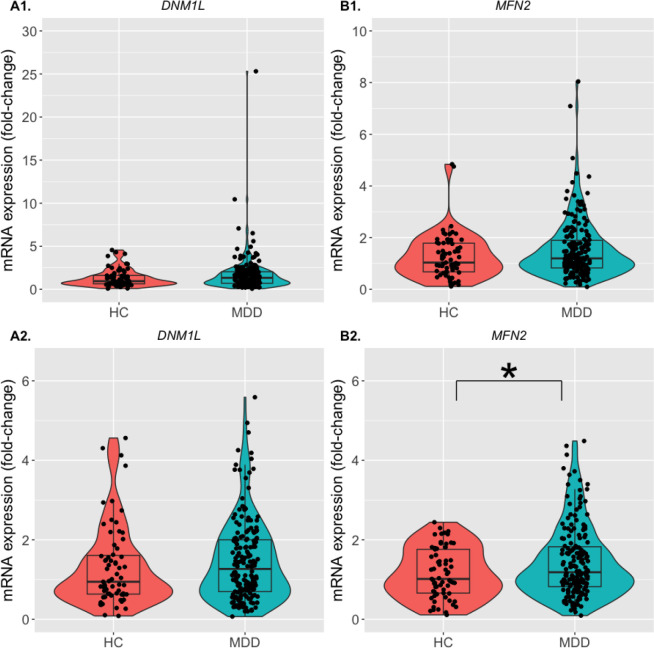
Expression of mitochondrial biogenesis genes in the MDD group compared to the HC group. The violin plots with superimposed box plots and individual data points indicating the fold-change of mRNA expression in the **(A1, A2)** *DNM1L* (left panel) and **(B1, B2)** *MFN2* (right panel) genes for the mitochondrial biogenesis. The HC group is represented by neon orange, and the MDD group is represented by teal. The limits of the boxes indicate the 25^th^ and 75^th^ percentile, while the horizontal line inside the boxes represent the median. The upper and lower limits of the vertical lines from the boxes indicate the 95^th^ percentile of the data. The y-axes indicate the fold-change of the genes’ mRNA expression compared to the respective reference gene (*GAPDH*) in the HC and the MDD groups. Upper panel (**A1, B1**) show all data points, while the lower panel (**A2, B2**) show data points excluding the outliers, which have been defined as data points > 3SD from the mean. The asterisk indicates statistically significant difference between the two groups (HC vs. MDD). *p < 0.05 after linear regression using age, sex, BMI, and medication status as covariates. HC: Healthy control; MDD: Major depressive disorder.

After controlling for age, sex, BMI, and medication status, there was a significant increase in the expression of the inflammasome genes, *NLRC4* (*p* = 0.040, uncorrected) and *NLRP3* (p = 0.035, uncorrected), but not *ASC* (*p* > 0.05), in the MDD group compared to the HC group (Fig. 1; Table 1). However, after FDR correction *NLRC4* and *NLRP3* trended significant (*p’s* = 0.064, corrected; Table 1). The expression of all three ER stress genes (*XBP1u, XBP1s, ATF4*) was significantly increased in the MDD group compared to the HC group (*p* = 0.007, *p* = 0.005, *p* = 0.006, respectively, uncorrected; Fig. 2; Table 1), and the results remained significant after FDR correction for multiple comparison (*p’*s = 0.019, corrected; Table 1). However, there was no significant difference in the expression of the mitochondrial biogenesis genes (*DNM1L*, *MFN2*) between the groups (*p* > 0.05; Fig. 3; Table 1).

In sensitivity analyses, which excluded the outliers (defined as > 3SD of the mean), the expression of the inflammasome genes, *NLRC4* and *NLRP3*, were no longer significantly different between the groups (Fig. 1; Table 1). All three ER stress genes (*XBP1u, XBP1s, ATF4*) remained significantly increased in the MDD group compared to the HC group (*p* = 0.045, 0.005, 0.025, respectively, uncorrected; Fig. 2; Table 1), but only *XBP1s* (*p* = 0.040, corrected; Table 1) remained significant after FDR correction. Additionally, sensitivity analysis revealed that the expression of the mitochondrial biogenesis gene, *MFN2*, was significantly increased in the MDD group compared to the HC group (*p* = 0.044, uncorrected; *p* > 0.05, corrected, Fig. 3; Table 1).

**Figure 4:**
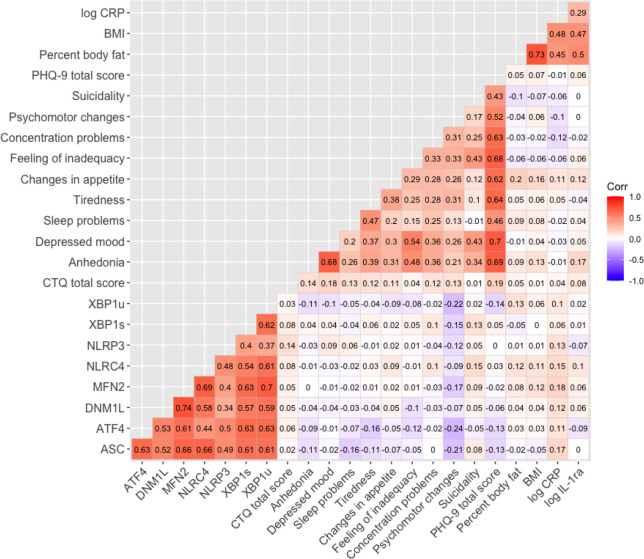
Correlations between symptoms, immunometabolic parameters, and mRNA expression within the MDD group. The correlogram shows the Spearman’s correlation coefficients for MDD participants. The fold-change of the mRNA expression for the inflammasome activation (*NLRC4, NLRP3, ASC*), ER stress (*XBP1u, XBP1s, ATF4*), and mitochondrial biogenesis (*DNM1L, MFN2*) are correlated with the CTQ total score, PHQ-9 scores (individual and total), percent body fat, BMI, CRP and IL-1ra. The color intensities represent the absolute values of correlation coefficients (r_s_). CTQ: Childhood Trauma Questionnaire; PHQ-9: Patient Health Questionnaire; CRP: C-reactive protein; IL-1ra: Interleukin-1 receptor antagonist. The r_s_ and *p* values are shown in the Supplementary Table 1.

We next tested whether the individual items of the PHQ-9 were associated with changes in the activity of the inflammasome, ER stress, and mitochondrial biogenesis genes in PBMCs. The PHQ-9 item, sleep problems, was significantly negatively correlated with the mRNA expression of the inflammasome gene *ASC* (rs = −0.157; *p* = 0.043); tiredness was significantly negatively correlated with the ER stress gene, *ATF4* (rs = –0.163; *p* = 0.037); psychomotor changes were negatively correlated with the inflammasome gene, *ASC* (rs = −0.212; *p* = 0.006), the ER stress genes *XBP1u* (rs = −0.219; *p* = 0.005), *XBP1s* (rs = − 0.151; *p* = 0.053), and *ATF4* (rs = −0.236; *p* = 0.002), and the mitochondrial biogenesis gene, *MFN2* (rs = –0.166; *p* = 0.033) (Fig. 4; Table 2). There were no significant correlations between gene expression and CTQ total score, percent body fat, BMI, or IL-1ra; however, CRP was significantly correlated with expression of the inflammasome gene *ASC* (rs = 0.173; *p* = 0.026) and the mitochondrial biogenesis gene *MFN2* (rs = 0.177; *p* = 0.022) (Fig. 4; Supplementary Table 1). Additionally, serum CRP levels showed a trend towards significance with the expression of the inflammasome gene, *NLRC4* (rs = 0.148; *p* = 0.059) (Fig. 4; Supplementary Table 1).

## DISCUSSION

This study compared the expression of genes involved in ER stress, inflammasome activity, and mitochondrial biogenesis in participants with MDD and HCs. To our knowledge, this is the first study to investigate the coordinated interplay of these organelles in the pathology of MDD. The most robust finding was an increase in mRNA transcripts of three representative genes of the UPR, i.e., *XBP1u, XBP1s*, and *ATF4*. The results remained significant with and without outliers as well as after FDR correction. To our knowledge there are only three published human studies that have investigated the link between alterations in ER homeostasis and the pathophysiology of MDD. Bown *et al*. initially reported increased levels of three ER stress-linked proteins (glucose regulated protein 78 kDa (GRP78), glucose regulated protein 94 kDa (GRP94), and calreticulin) in the temporal cortex of samples with MDD (n = 15) who died by suicide^62^. Consistent with these data, the expression of *GRP78, GRP94*, and *ATF4*, was found to be increased in the dorsolateral prefrontal cortex of an independent MDD sample (n = 43) who died by suicide^63^. The third study focused on the expression of UPR-associated genes in leukocytes, finding that a sample of MDD participants (n = 18) showed greater expression of *GRP78*, ER degradation-enhancing alpha-mannosidase-like 1 (*EDEM1*), C/EBP homologous protein (*CHOP*), and *XBP1* relative to controls (n = 18)^64^. Our results are largely consistent with these studies although we did not observe significant correlations between gene expression and the suicidality item of the PHQ-9, suggesting that the difference in the previous findings may be due to the effects of depression rather than suicide *per se*.

The coordinated activation of the UPR serves as a mechanism to rectify ER stress by augmenting protein folding capacity, promoting protein degradation, modulating global translation rates, and fine-tuning cellular metabolism^36^. However, as noted above, chronic, unresolved ER stress can lead to enduring activation of the UPR with serious long-term consequences including the propagation of toxic protein aggregates and neuronal degeneration when present in neuronal cells^40–42^. Because we only measured gene expression at one time point, we cannot draw definitive conclusions about the chronicity of the UPR activation in this study. Nevertheless, it is conceivable that a significant number of people with MDD display chronic ER stress in their immune cells which may have functional consequences. For instance, ER stress is known to modulate inflammatory regulators such as NF-κB and JNK-AP1, thereby upregulating the production of pro-inflammatory cytokines such as IL-6, IL-1β, and tumor necrosis factor (TNF)^65^. Moreover, ER stress can activate the NLRP3 inflammasome, a mechanism that is now hypothesized to be the pathological basis of various inflammatory diseases^65^. Of note, we observed significant correlations (r_s_ = 0.4–0.6; Supplementary Fig. 1) between all three inflammasome-related genes and the UPR genes in both the MDD and HC groups. To our knowledge, the relationship between NLRC4 and the UPR has not been investigated. The current data raise the possibility that ER stress may also activate the NLRC4 inflammasome.

The second major finding was the significant increase in the mRNA expression of the genes involved in inflammasome activity (*NLRC4* and *NLRP3*). These results were less robust as they trended significant after FDR correction and were not statistically significant after the exclusion of outliers. However, we note that the MDD-associated increase in inflammatory mediators typically observed in the literature is well known to be driven by a minority of individuals. As Miller and Raison^66^ write “It is a dirty little secret of sorts that the one-third or so of depressed individuals with elevated inflammation have been pulling all their noninflamed, depressed colleagues along with them in publication after publication, giving the world a slightly misguided sense that depression—as a whole—is driven by increased inflammation”. Thus, it is likely that rather than being indicative of measurement error, the small subset of MDD participants with outlying *NLRC4* and *NLRP3* expression levels may reflect the true biological picture in this population.

To our knowledge, this is the first paper to report an increase in NLRC4 expression in MDD. The NLRC4 inflammasome is primarily activated by intracellular pathogens, including viruses and gram-negative bacterial pathogens such as *Salmonella, Legionella, Pseudomonas*, and *Shigella*^10, 11, 67^. The bacterial flagellin and type III secretion system (T3SS) is known to activate the NLRC4 inflammasome^68^, and interestingly, a flagellin-TLR5 interaction allows us to differentiate between commensal-derived flagellin and pathogen-derived flagellin originating from the gut^69^. The gut microbiome has been implicated in MDD^70, 71^ and it is conceivable that NLRC4 inflammasomes activated by gut bacterial flagellin may play an unrecognized role in this relationship. In addition, the increase in *NLRC4* expression raises the possibility of a viral etiology as we have previously hypothesized^72–75^, but it is also possible that the effect is driven by ER stress.

The NLRP3 inflammasome, which is activated by a wide variety of endogenous danger signals and cellular stressors^12–14^, has previously been implicated in MDD^25–28^. Two of these studies also reported concomitant elevations in circulating IL-1β and/or IL-18^25, 26^. Similarly, Nemeroff and colleagues reported elevated protein levels of caspase-1, ASC-1, and IL-18 in MDD participants (n = 24) compared to HC subjects (n = 24)^76^. We used IL-1ra, the anti-inflammatory antagonist of the interleukin-1 family of pro-inflammatory cytokines, as a surrogate marker of IL-1β since in the T-1000 cohort most individuals have IL-1β concentrations that are below detectable limit. However, it is possible that IL-1ra concentrations do not adequately reflect inflammasome-induced increases in IL-1β as factors other than IL-1β concentration (i.e., IgG, other cytokines, and pathogen-associated molecular patterns) can also influence IL-1ra^77^. Alternatively, the increased expression of *NLRC4* and *NLRP3* may not be indicative of inflammasome activation since it is known that mRNA concentrations are not always reflective of protein concentrations because of changes in translational efficiency and/or post-translational modifications^78^.

There were no significant group differences in the expression of the mitochondrial biogenesis genes, *MFN2* and *DNM1L*, although after exclusion of outliers, the mRNA levels of *MFN2* were significantly increased in the MDD group compared to the HC group. Previous studies have investigated the bioenergetic functions of mitochondria from the PBMCs of MDD participants and have reported conflicting results. For instance, one study found significant impairment of mitochondrial bioenergetic functions indicated by reduced mitochondrial respiration, ATP turnover-related respiration and spare respiratory capacity, and mitochondrial coupling efficiency in MDD patients^79^. However, a more recent study did not find any difference in the mitochondrial respiration and overall mitochondrial health index measured from the PBMCs of MDD participants^80^. Nevertheless, the phenomenon of mitochondrial biogenesis *per se* has been less studied in the context of MDD. To our knowledge, *DNM1L* expression has not been previously investigated in MDD, and only one study examined the expression of *MFN2* in the PBMCs of a sample with MDD, where *MFN2* was also found to be increased in individuals with MDD (n = 77) compared with healthy controls (HCs; n = 24)^55^. Quevedo and colleagues also observed that MFN2 mRNA levels were greater in the MDD group with CRP concentrations in the top 50% of the distribution compared with the MDD group with CRP concentrations in the bottom 50%^55^. In our sample, CRP concentrations were positively and significantly associated with *MFN2* expression in the MDD group (rs = 0.177, p = 0.022; Supplementary Table 1).

The literature is suggestive of a bidirectional relationship between mitochondrial biogenesis and ER stress. Pre-clinical *in vitro* and *in vivo* studies show that ER stress upregulates MFN2 both at the mRNA transcript and protein levels, and MFN2 in turn protects cells from ER stress^81^. Consistent with these data, we observed significant positive correlations (rs = 0.6–0.7) between *MFN2* and *XBP1u, XBP1s*, and *ATF4* expression (Supplementary Fig. 1). MFN2 is also known to modulate the inflammasome-dependent innate immune response and immunometabolic effects^82^, including the NLRP3 inflammasome^4, 83^. Here we observed significant positive correlations between *MFN2* and both *NLRP3* (r_s_ = 0.4) and *NLRC4* (r_s_ = 0.69) expression (Supplementary Fig. 1).

This study has several limitations. First, like other gene expression studies we are unable to draw definitive conclusions about inflammasome activation, ER function, and mitochondrial biogenesis from mRNA transcripts alone. Protein measurements and functional assays of these organelles would provide important complementary information. A second limitation is that we did not measure IL-1β and IL-18 levels. Third, because we measured mRNA expression from PBMCs we cannot determine which type of immune cells are driving the reported group differences. Finally, because of our focus on the inter-relationship between the three organelles, we measured the expression of select representative genes from each domain. It is possible that different results would have been obtained with other candidate genes (although we note that correlations between the selected genes within each domain were significant).

In sum, we observed robust evidence of greater expression of UPR-linked genes in MDD compared with HC. We also found evidence of increased expression of the NLRP3 and NLRC4 inflammasomes and to a lesser extent, increased mitochondrial biogenesis in the MDD sample. Given that ER stress is known to upregulate NLRP3, NLRC4, and MFN2, it is conceivable that ER stress is the underlying cause of the intracellular changes in MDD. Nevertheless, our study design does not allow us to determine which organelle is the primary driver of these alterations in gene expression. Rather our findings underscore the potential importance of intracellular dysfunction in MDD and suggest that future studies take a “holistic” approach to the investigation of these putative abnormalities.

## Figures and Tables

**Table 1.** Demographics, clinical characteristics, and RT-qPCR results of the study participants.

		HC	MDD	p		SMD
**n**		67	186			
**Age (years)**	mean (SD)	31.32 (11.16)	34.38 (11.28)	0.058		0.272
**Sex**	male (%)	31 (46.3)	58 (31.2)	**0.039** *		0.313
**Medication status**	unmedicated (%)	60 (89.6)	72 (38.7)	**<0.001** ***		1.250
**Race and ethnicity**				0.306		0.358
**Asian**	number (%)	3 (5.0)	2 (1.2)			
**Black**	number (%)	2 (3.3)	13 (7.6)			
**Hispanic**	number (%)	3 (5.0)	8 (4.7)			
**Native American**	number (%)	6 (10.0)	26 (15.3)			
**White**	number (%)	45 (75.0)	115 (67.6)			
**Other**	number (%)	1 (1.7)	6 (3.5)			
**BMI (kg/m^2^)**	mean (SD)	27.14 (5.69)	28.63 (5.45)	0.06		0.266
**Percent body fat**	mean (SD)	30.34 (10.86)	35.75 (10.13)	**0.001** **		0.515
**CTQ: total score**	mean (SD)	32.98 (9.00)	48.35 (19.66)	**<0.001** ***		1.005
**PHQ-9: anhedonia**	mean (SD)	0.03 (0.18)	1.50 (0.82)	**<0.001** ***		2.482
**PHQ-9: depressed mood**	mean (SD)	0.02 (0.13)	1.51 (0.80)	**<0.001** ***		2.596
**PHQ-9: sleep problems**	mean (SD)	0.15 (0.36)	2.05 (0.93)	**<0.001** ***		2.689
**PHQ-9: tiredness**	mean (SD)	0.28 (0.45)	2.12 (0.84)	**<0.001** ***		2.725
**PHQ-9: changes in appetite**	mean (SD)	0.07 (0.25)	1.50 (1.06)	**<0.001** ***		1.850
**PHQ-9: feeling of inadequacy**	mean (SD)	0.02 (0.13)	1.81 (1.00)	**<0.001** ***		2.517
**PHQ-9: concentration problems**	mean (SD)	0.07 (0.31)	1.41 (0.99)	**<0.001** ***		1.827
**PHQ-9: psychomotor changes**	mean (SD)	0.03 (0.18)	0.80 (0.85)	**<0.001** ***		1.247
**PHQ-9: suicidality**	mean (SD)	0.00 (0.00)	0.41 (0.69)	**<0.001** ***		0.832
**PHQ-9: total score**	mean (SD)	0.67 (1.17)	13.11 (5.00)	**<0.001** ***		3.424
**log CRP**	mean (SD)	0.81 (2.43)	0.79 (2.02)	0.950		0.009
**log IL-1ra**	mean (SD)	5.58 (1.27)	5.89 (0.97)	**0.052**		0.276
						
**RT-qPCR data**						
**DC_T_ (mean (SD))**						
**1. Inflammasome activation genes**						
	*NLRC4*	5.93 (0.94)	5.60 (1.01)	0.021*		0.336
	*NLRP3*	4.53 (0.91)	4.42 (1.32)	0.550		0.092
	*ASC*	3.34 (0.97)	3.21 (1.39)	0.482		0.108
**2. ER stress genes**						
	*XBP1u*	4.08 (1.73)	3.50 (0.98)	**0.001** **		0.410
	*XBP1s*	4.31 (1.27)	3.79 (1.01)	**0.001** **		0.454
	*ATF4*	2.60 (1.09)	2.17 (1.08)	**0.007** **		0.390
**3. Mitochondrial biogenesis genes**						
	*DNM1L*	5.60 (1.15)	5.35 (1.19)	0.142		0.212
	*MFN2*	5.70 (1.08)	5.42 (1.01)	0.061		0.264
						
**DDC_T_ (mean (SD))**						
**1. Inflammasome activation genes**						
	*NLRC4*	0.00 (0.94)	−0.33 (1.01)	**0.021** *		0.336
	*NLRP3*	0.00 (0.91)	−0.10 (1.32)	0.550		0.092
	*ASC*	0.00 (0.97)	−0.13 (1.39)	0.482		0.108
**2. ER stress genes**						
	*XBP1u*	0.00 (1.73)	−0.58 (0.98)	**0.001** **		0.410
	*XBP1s*	0.00 (1.27)	−0.52 (1.01)	**0.001** **		0.454
	*ATF4*	0.00 (1.09)	−0.42 (1.08)	**0.007** **		0.390
**3. Mitochondrial biogenesis genes**						
	*DNM1L*	0.00 (1.15)	−0.25 (1.19)	0.142		0.212
	*MFN2*	0.00 (1.08)	−0.28 (1.01)	0.061		0.264
						
**Fold-change = 2^−DDCT^ (mean (SD))**						
				**p (after multiple regression)**	**p (with FDR correction)**	
**1. Inflammasome activation genes**						
	**All data points**					
	*NLRC4*	1.21 (0.73)	1.58 (1.13)	**0.040** *	0.064	0.389
	*NLRP3*	1.20 (0.76)	1.48 (1.63)	**0.035** *	0.064	0.214
	*ASC*	1.21 (0.70)	1.40 (0.92)	0.118	0.157	0.231
	**Excluding outliers**					
	*NLRC4*	1.21 (0.73)	1.41 (0.79)	0.182	0.208	0.264
	*NLRP3*	1.20 (0.76)	1.18 (0.70)	0.127	0.169	0.031
	*ASC*	1.21 (0.70)	1.33 (0.78)	0.126	0.169	0.158
**2. ER stress genes**						
	**All data points**					
	*XBP1u*	1.36 (0.85)	1.84 (1.25)	**0.007** **	**0.019** *	0.445
	*XBP1s*	1.35 (1.04)	1.80 (1.26)	**0.005** **	**0.019** *	0.396
	*ATF4*	1.29 (0.95)	1.74 (1.37)	**0.006** **	**0.019** *	0.386
	**Excluding outliers**					
	*XBP1u*	1.36 (0.85)	1.67 (0.88)	**0.045** *	0.090	0.349
	*XBP1s*	1.23 (0.80)	1.68 (1.01)	**0.005** **	**0.040** *	0.495
	*ATF4*	1.13 (0.64)	1.53 (0.92)	**0.025** **	0.090	0.498
**3. Mitochondrial biogenesis genes**						
	**All data points**					
	*DNM1L*	1.32 (1.02)	1.70 (2.19)	0.365	0.365	0.220
	*MFN2*	1.27 (0.88)	1.52 (1.13)	0.228	0.261	0.254
	**Excluding outliers**					
	*DNM1L*	1.32 (1.02)	1.42 (0.96)	0.231	0.231	0.102
	*MFN2*	1.16 (0.63)	1.39 (0.82)	**0.044** *	0.090	0.316

***Note:*** BMI: Body mass index; CRP: C- reactive protein (mg/dL); C_T_: Cycle threshold; CTQ: Childhood Trauma Questionnaire; DC_T_: Difference of C_T_ values between the target gene and reference (*GAPDH*) gene; DDC_T_: Difference between average C_T_ values of HC group and DC_T_ of the respective target genes; PHQ-9: Patient Health Questionnaire 9; HC: Healthy control; IL-1ra: Interleukin-1 receptor antagonist; MDD: Major depressive disorder; RT-qPCR: Reverse transcription – quantitative polymerase chain reaction; SMD: Standardized mean difference. The data points > 3SD from the mean were considered outliers.

* *p* < 0.05

** *p* < 0.01

*** *p* < 0.001: Calculated using c^2^ test for categorical data and two-tailed *t*-test for numerical data. Statistics for the mRNA fold-change are from a linear regression using the lm model with age, sex, BMI, and medication status as covariates.

**Table 2.** Correlations between mRNA expression and depressive symptoms in the MDD group.

		Depressive symptoms (PHQ-9)									PHQ-9 total score
		*Anhedonia*	*Depressed mood*	*Sleep problems*	*Tiredness*	*Changes in appetite*	*Feelings of inadequacy*	*Concentration problems*	*Psychomotor changes*	*Suicidality*	
**Inflammasome genes**											
*NLRC4*	**r_s_**	−0.014	−0.029	−0.016	0.030	0.085	−0.010	0.099	−0.090	0.145	0.025
	***p***	0.861	0.710	0.836	0.707	0.277	0.894	0.208	0.253	0.064	0.746
											
*NLRP3*	**r_s_**	−0.026	0.086	0.056	−0.013	0.016	0.007	−0.038	−0.125	0.052	−0.002
	***p***	0.740	0.268	0.475	0.872	0.839	0.924	0.623	0.108	0.506	0.975
											
*ASC*	**r_s_**	−0.113	−0.017	−0.157	−0.113	−0.068	−0.052	0.003	−0.212	0.078	−0.113
	***p***	0.145	0.830	**0.043** *	0.147	0.385	0.505	0.970	**0.006** **	0.316	0.097
**ER stress genes**											
*XBP1u*	**r_s_**	−0.111	−0.096	−0.055	−0.035	−0.092	−0.078	−0.015	−0.219	0.023	−0.136
	***p***	0.157	0.219	0.485	0.655	0.241	0.318	0.844	**0.005** **	0.767	0.082
											
*XBP1s*	**r_s_**	0.040	0.038	−0.045	0.055	0.019	0.054	0.095	−0.151	0.132	0.051
	***p***	0.612	0.624	0.569	0.479	0.804	0.489	0.222	**0.053**	0.090	0.510
											
*ATF4*	**r_s_**	−0.085	−0.014	−0.070	−0.163	−0.052	−0.117	−0.023	−0.236	−0.046	−0.135
	***p***	0.278	0.863	0.372	**0.037** *	0.510	0.137	0.771	**0.002** **	0.559	0.086
**Mitochondrial biogenesis genes**											
*DNM1L*	**r_s_**	−0.042	−0.044	−0.030	−0.037	0.045	−0.097	−0.034	−0.070	0.050	−0.057
	***p***	0.592	0.572	0.698	0.636	0.564	0.212	0.667	0.370	0.520	0.468
											
*MFN2*	**r_s_**	0.003	−0.011	−0.025	0.014	0.022	0.011	−0.033	−0.166	0.085	−0.021
	***p***	0.973	0.887	0.754	0.862	0.783	0.892	0.670	**0.033** *	0.274	0.787

***Note:*** PHQ-9: Patient Health Questionnaire 9 was used for assessment of individual depressive symptoms. Spearman’s correlation analysis revealed that **psychomotor changes** was significantly negatively correlated with mRNA expression of ER stress genes (*XBP1u*, *XBP1s*, *ATF4*), mitochondrial biogenesis gene (*MFN2*) and inflammasome activation gene (*ASC*); **sleep problems** was significantly negatively correlated with mRNA expression of inflammasome activation gene (*ASC*); and **tiredness** was significantly negatively correlated with mRNA expression of ER stress gene (*ATF4*).

* *p* < 0.05

** *p* < 0.01: Calculated using Spearman’s correlation tests.
